# Biofilm formation by virulent and non-virulent strains of *Haemophilus parasuis*

**DOI:** 10.1186/s13567-014-0104-9

**Published:** 2014-11-27

**Authors:** Bernardo Bello-Ortí, Vincent Deslandes, Yannick DN Tremblay, Josée Labrie, Kate J Howell, Alexander W Tucker, Duncan J Maskell, Virginia Aragon, Mario Jacques

**Affiliations:** Centre de Recerca en Sanitat Animal (CReSA), UAB-IRTA, Campus de la Universitat Autònoma de Barcelona, 08193 Bellaterra, Cerdanyola del Vallès, Spain; Groupe de recherche sur les maladies infectieuses du porc, Faculté de médecine vétérinaire, Université de Montréal, St-Hyacinthe, Québec, J2S 7C6 Canada; Department of Veterinary Medicine, University of Cambridge, Cambridge, UK; Institut de Recerca i Tecnologia Agroalimentàries (IRTA), Barcelona, Spain

## Abstract

*Haemophilus parasuis* is a commensal bacterium of the upper respiratory tract of healthy pigs. It is also the etiological agent of Glässer’s disease, a systemic disease characterized by polyarthritis, fibrinous polyserositis and meningitis, which causes high morbidity and mortality in piglets. The aim of this study was to evaluate biofilm formation by well-characterized virulent and non-virulent strains of *H. parasuis*. We observed that non-virulent strains isolated from the nasal cavities of healthy pigs formed significantly (*p* < 0.05) more biofilms than virulent strains isolated from lesions of pigs with Glässer’s disease. These differences were observed when biofilms were formed in microtiter plates under static conditions or formed in the presence of shear force in a drip-flow apparatus or a microfluidic system. Confocal laser scanning microscopy using different fluorescent probes on a representative subset of strains indicated that the biofilm matrix contains poly-N-acetylglucosamine, proteins and eDNA. The biofilm matrix was highly sensitive to degradation by proteinase K. Comparison of transcriptional profiles of biofilm and planktonic cells of the non-virulent *H. parasuis* F9 strain revealed a significant number of up-regulated membrane-related genes in biofilms, and genes previously identified in *Actinobacillus pleuropneumoniae* biofilms. Our data indicate that non-virulent strains of *H. parasuis* have the ability to form robust biofilms in contrast to virulent, systemic strains. Biofilm formation might therefore allow the non-virulent strains to colonize and persist in the upper respiratory tract of pigs. Conversely, the planktonic state of the virulent strains might allow them to disseminate within the host.

**Electronic supplementary material:**

The online version of this article (doi:10.1186/s13567-014-0104-9) contains supplementary material, which is available to authorized users.

## Introduction

*Haemophilus parasuis* is a Gram-negative bacterium and a commensal organism of the upper respiratory tract of healthy pigs. It is also the etiological agent of Glässer’s disease, a systemic disease characterized by polyarthritis, fibrinous polyserositis and meningitis, which causes high morbidity and mortality in piglets [1]. Glasser’s disease is recognized as one of the main causes of economic loss in the pig industry.

The heterogeneity among *H. parasuis* strains has been demonstrated by multiple methods, including multilocus sequence typing (MLST) which has shown a genetic lineage associated with polyserositis (cluster A) and another lineage associated with nasal colonization (cluster C) [2]. Serum resistance [3], phagocytosis resistance [4], and invasion of endothelial cells [5,6] have been associated with virulence of *H. parasuis*. Some putative virulence factors have been reported [7,8]. Those include the capsule [4], lipooligosaccharide (LOS) [9-11] and other genes involved in polysaccharide production, such as *galE* and *galU*, which have been associated with serum resistance and biofilm production [12]. Genes involved in sialic acid utilization were identified as potential virulence factors [13]. The sialytransferase gene *lsgB* was predominantly present in systemic isolates and not in nasal isolates, and sialylation of the LOS was observed in the virulent strain *H. parasuis* Nagasaki. In addition, a family of trimeric autotransporters, designated virulence associated trimeric autotransporters (VtaA) has been described [14,15] and these can be divided into three groups based on their translocation domains [14]. Group 3 *vtaA* gene is highly conserved among invasive and non-invasive strains, whilst groups 1 and 2 *vtaA* genes were detected only in virulent strains.

Bacterial biofilms are structured clusters of bacterial cells enclosed in a self-produced polymer matrix that are attached to a surface [16-18]. Bacteria can adhere to biotic surfaces (e.g. cells at the mucous layer) as well as abiotic surfaces (e.g. floor or equipment found at a farm). The polymer matrix is often composed of exopolysaccharides, proteins and nucleic acids. The biofilm protects bacteria from hostile environmental conditions. Bacteria within a biofilm can resist attack from the host immune response, and are less sensitive than planktonic cells to desiccation and to the action of biocides. Biofilm formation by nasal strains of *H. parasuis* has been previously reported [19]. Although the role of biofilm in *H. parasuis* pathogenesis is not clear, expression of genes with putative function in biofilm formation was detected during pulmonary infection [20]. The aims of this study were to compare biofilm formation by well-characterized virulent and non-virulent strains of *H. parasuis* and to analyse the gene expression during biofilm growth. Such analyses would help explore the possible role of biofilms in the pathogenesis of *H. parasuis*.

## Materials and methods

### Bacterial strains

The *H. parasuis* strains used in this study and their main phenotypic and genotypic characteristics are listed in Table 1. Bacteria were grown on Brain Heart Infusion (BHI; Oxoid Ltd, Basingstoke, Hampshire, UK) agar containing 10 μg/mL NAD or on chocolate agar (Biomerieux, Madrid, Spain). Plates were incubated overnight at 37 °C with 5% CO_2_.

**Table 1:** ***Haemophilus parasuis*** **strains used in the present study**

**Strains**	**Susceptibility** ^**1**^ **to**		***lsgB*** ^**2**^	**group1** ***vtaA*** ^**2**^	**MLST** ^**3**^	**Serovar**
	**Serum**	**Phagocytosis**				
**Nasal strains**						
F9	S	S	-	-	C	6
ND14-1	S	S	-	-	C	7
SC14-1	S	S	-	-	C	15
MU21-2	S	S	-	-	C	7
SL3-2	S	S	-	+	B	10
**Strains from lesions**						
Nagasaki	R	R	+	+	A	5
P015/96	R	R	+	+	A	5
ER-6P	R	R	+	+	A	15
264/99	R	R	+	+	A	10
IT29205	R	R	+	+	A	4
2725	S	R	+	+	A	10
373/03A	I	R	-	+	A	7
PV1-12	S	R	-	+	B	15
9904108	S	R	-	-	C	4

^1^(S) sensitive; (I) intermediate; (R) resistant.

^2^(+) gene present; (−) gene absent.

^3^Cluster associated with isolates from systemic lesions (A); with nasal isolation (C); or cluster with no statistical association with clinical origin (B) [2].

### Biofilm assays

#### Static conditions

Biofilms were cultured in 96-well microtiter plates as described by Wu et al. [21], with some modifications. Briefly, colonies from overnight agar cultures were resuspended in BHI-NAD containing 5 μg/mL NAD, and the suspension was aliquoted (100 μL) in triplicate in a flat-bottom 96-well polystyrene plate (Costar® 3599, Corning, NY, USA) and incubated for 40 h at 37 °C. Wells containing sterile broth were used as negative control. Following incubation, biofilms were treated as described before [22] with some modifications. Briefly, the liquid medium was removed using a vacuum and unattached cells were removed by immersing the plate once in MilliQ water. The water was removed with a vacuum and excess water was removed by inverting plates onto a paper towel. Biofilms were then stained with 0.1% (w/v) crystal violet for 2 min. Biofilms were washed once with distilled water and then dried at 37 °C for 15 min. The stain was then released with 100 μL of 70% (v/v) ethanol and the amount of released stain was quantified by measuring the absorbance at 590 nm with a microplate reader (Powerwave; BioTek Instruments, Winooski, VT, USA).

#### Shear force conditions

Biofilms were cultured in a drip-flow apparatus (DFR 110 Biofilm Reactor, BioSurface Technologies Corp. Bozeman, MT, USA) as described by Goeres et al. [23] with some modifications [24]. Briefly, colonies of *H. parasuis* strains F9, MU21-2, ER-6P or Nagasaki on BHI-NAD agar were resuspended into 13 mL of fresh BHI-NAD to an OD_600_ of 0.1 and 12.5 mL of this inoculum was transferred into a channel containing a glass slide (Catalogue #48300-025, VWR, Ville Mont-Royal, QC, Canada). The apparatus was incubated for 24 h at 37 °C with 5% CO_2_ to allow the biofilm to form under static conditions. The apparatus legs were then attached to create a 10° downward slope. The apparatus was then connected to the nutrient system containing pre-warmed (37 °C) BHI-NAD. The flow (~25 mL per hour per channel) of the medium was then initiated and maintained for 24 h at 37 °C. After 24 h, the glass slide was removed and gently washed once with sterile MiliQ water. The biofilms were resuspended in 1.5 mL of MiliQ water, centrifuged and dried with a DNA 120 Speed Vac® (Thermo Scientific, Ottawa, ON, Canada). The weight of dry biofilms was then measured.

Biofilms were also cultured in a microfluidic system. Growth of biofilms in the BioFlux 200 device (Fluxion Biosciences, South San Francisco, CA, USA) was adapted from Benoit et al. [25] and the manufacturer’s recommendations. Briefly, colonies of *H. parasuis* strains F9, MU21-2, ER-6P or Nagasaki on BHI-NAD agar were resuspended into 2.5 mL of fresh BHI-NAD to an OD_600_ of 1.0 and this suspension was serially diluted to OD_600_ of 0.5 and 0.25. The microfluidic channels were wetted with BHI-NAD and were then inoculated with different bacterial suspensions (OD_600_ 1.0, 0.5 or 0.25). The microfluidic plate was incubated for 2 h at 37 °C to allow bacteria to bind to the surface. The flow of fresh medium was then initiated and was set from 0.5 dyne/cm^2^ to 1.0 dyne/cm^2^. Growth of the biofilms was monitored for up to 24 h and, in some cases, fresh medium was added and the “waste” outlet was emptied to ensure that wells would not dry or spill. Images of BioFlux biofilms were obtained using an inverted fluorescence microscope (Olympus CKX41, Markham, ON, Canada), a digital camera (Retiga EX; Q Imaging, Surrey, BC, Canada), and the software provided with the BioFlux 200 device.

### Confocal laser scanning microscopy (CLSM)

Biofilms were prepared under static conditions as described above and were stained with FilmTracer™ FM®1-43 Green biofilm cell stain (Molecular Probes, Eugene, OR, USA) according to manufacturer’s instructions. To determine the composition of the biofilm matrix, biofilms were stained with Wheat Germ Agglutinin (WGA-Oregon Green 488, Molecular Probes; binds to N-acetyl-D-glucosamine and N-acetylneuraminic acid residues), FilmTracer™ SYPRO® Ruby biofilm matrix stain (Molecular Probes; labels most classes of proteins) or BOBO™-3 iodide (Molecular Probes; stains extracellular DNA) according to manufacturer’s instructions. After a 30 min incubation at room temperature, the fluorescent marker solution was removed, biofilms were washed with water and the wells were then filled with 100 μL of water or PBS for WGA-stained biofilms. Stained biofilms were visualized by CLSM (Olympus FV1000 IX81, Markham, ON, Canada).

### Dispersion of biofilm by enzymatic treatments

A biofilm dispersion assay was performed as described previously [26]. Briefly, biofilms were grown under static conditions as described above, and after the 40 h incubation, 50 μL of DNase I (500 μg/mL in 150 mM NaCl, 1 mM CaCl_2_), 50 μL of dispersin B (100 μg/mL in PBS; Kane Biotech Inc., Winnipeg, MB, Canada), or 50 μL of proteinase K (500 μg/mL in 50 mM Tris–HCl pH 7.5, 1 mM CaCl_2_) were added directly to the biofilms. Control wells were treated with 50 μL of the buffer without the enzyme. Wells treated with dispersin B were incubated for 5 min at 37 °C, and those treated with proteinase K or DNase I were incubated for 1 h at 37 °C. After the treatments, the biofilms were stained with crystal violet as described above.

### Effect of fibrinogen and fibronectin on biofilm formation

Biofilms were prepared under static conditions as described above with some modifications. Prior to inoculation, various concentrations of fibrinogen (bovine, porcine or human) (0.5, 1, 2 and 5 mg/mL) or fibronectin (human) (0.5, 1 and 2 mg/mL) were added to the biofilm medium. Plates were prepared in duplicate for each experiment: one plate was used to measure biofilm formation and the other plate was used to measure growth of the bacteria in the presence of proteins. Both plates were incubated and one plate was stained as described before. The unstained replicate plate was used to evaluate growth by measuring the absorbance at 600 nm.

### Genome sequencing and assembly

Genomic DNA of the high biofilm producer strain F9 was prepared using the Blood and Tissue DNeasy kit (Qiagen) according to the manufacturer’s instructions. Illumina libraries were prepared using 500 ng of genomic DNA and modified Illumina protocols [27,28]. Paired-end sequencing was performed on an Illumina HiSeq 2000 analyzer for 75 cycles at the Wellcome Trust Sanger Institute (Hinxton, Cambridge, UK). The fastq reads were mapped to the complete SH0165 genome using Stampy to check for quality and purity of the sequence before any analyses [29,30].

A custom-made bioinformatics pipeline was used to assemble the draft genome sequence. Cutadapt was used to remove the adaptor sequences from the sequence reads that were previously introduced during the library preparation [31]. Any undetermined nucleotides (N’s) were removed from reads and the program sickle [32] was used to trim low-quality sequences found at the ends of paired-end sequence reads resulting in a minimum length of 31 bp, using the program’s default quality thresholds for the reads. Next, a custom Perl script was used to screen out low quality fastq reads and produce a single fastq file containing the good paired reads, and a separate file containing good quality single reads. Finally, we used Velvet [33] and VelvetOptimiser 2.2.0 [34] to assemble the fastq files into the de-novo genome assembly, made up of contiguous sequences (contigs). Assembly parameters were optimised to produce the highest quality assembly (i.e. highest n50 value) using VelvetOptimiser, which runs through all possible k-mer values from 19 to 71 in increments of 2. The draft genome sequence was annotated using the automatic annotation software Prokka [35], including the rfam option. This Whole Genome Shogun project has been deposited [DDBJ/EMBL/GenBank: JHQI00000000]. The version described in this paper is version JHQI01000000.

### Transcriptomics analysis

The high biofilm producer strain F9 was chosen for transcriptomic analysis. Planktonic and biofilm samples were obtained under static conditions as described above with some modifications. Bacteria were cultured in 6-well microtiter plates and after 36 h, planktonic cultures were transferred to sterile tubes, whereas biofilm bacteria were collected by scraping the surface of the wells with lysis buffer (2% SDS in PBS). Bacteria from both samples, biofilm or planktonic, were recovered by centrifugation and pellets were used for RNA extraction. For comparison, F9 was grown with shaking (220 rpm) until the culture reached stationary phase and this culture was then processed for RNA extraction. This is considered to be the “stationary culture” sample.

To extract RNA, bacterial pellets were resuspended in 2% SDS, 16 mM EDTA, 10 mM Tris (pH 8.0) and incubated for 5 min at 100 °C. Afterwards, samples were processed by two hot acid phenol-chloroform extractions, followed by two chloroform/isoamyl alcohol extractions. RNA was then precipitated with 0.6 volumes of isopropanol, 0.1 volumes of 5 M ammonium acetate and 1 μL of glycogen. After centrifugation, the pellet was washed with 70% ethanol, dried and resuspended in warmed RNase-free water. To ensure that contaminating bacterial DNA was eliminated from the samples, treatment with RNase-free DNase (Qiagen) was performed. In addition, ribosomal RNA was eliminated with the Ribo-Zero rRNA removal kit (Epicentre Biotechnologies, Madison, WI, USA) following manufacturer instructions. PCR reactions using primers specific for *H. parasuis* 16S rRNA gene [36] were carried out to ensure that no bacterial DNA was left in the sample. Final RNA quality was verified with a Nanodrop spectrophotometer and the integrity was analyzed using Agilent Bioanalyzer 2100 (Agilent technologies). Libraries were generated using an Ion Torrent RNA-Seq v2 kit (Life Technologies) and sequenced using an Ion Torrent PGM (Life Technologies) with an Ion 318 chip (Life technologies) at the Centre for Research in Agricultural Genomics (CRAG, Campus de Bellaterra-UAB, Spain).

Bioinformatic analysis was performed following the count-based differential expression method. Briefly, reads quality control was performed using FastQC [37] and FASTX-Toolkit [38], and reads were then mapped to *H. parasuis* F9 genome using the recommended Torrent Mapping Alignment Program (TMAP) v3.4.1 with map2 setting [39]. Alignments were inspected using SAMtools [40] and Integrative Genomics Viewer (IGV) [41]. HTSeq v0.5.4p3 [42] was used for feature counting with intersection-nonempty setting and discarding non-protein coding CDS. Differentially expressed genes (DEGs) were identified with edgeR R package [43] with a 5% *P*-value cut-off, using an assigned dispersion value of 0.04.

To perform gene set enrichments, a custom Gene Ontology (GO) database was built. Protein coding genes were BLASTed using BLASTp (version 2.2.28) against the non-redundant NCBI database (April 2014), e value of 10^−3^ and keeping first 20 hits. GO terms were mapped to Blast hits using Blast2GO [44]. The most significantly up-regulated genes (*P* < 0.05) were identified as candidates for classic Fisher’s exact testing through Blast2GO. Tests were performed for biological process (BP), cellular component (CC) and molecular function (MF) with *P* < 0.05. Conservation of membrane-related genes among all 14 *H. parasuis* sequences available in GenBank [GenBank: APCA00000000.1, APBW00000000.1, ABKM00000000.2, APBX00000000.1, AZQU00000000.1, APBZ00000000.1, AOSU00000000.1, APBY00000000.1, APBV00000000.1, CP001321.1, APBT00000000.1, APBU00000000.1, APCB00000000.1, CP005384.1] was achieved as follows: whole F9 proteome was analyzed using Phobius [45] via Blast2GO, positively predicted membrane-related genes were blasted against the 14 *H. parasuis* genomic sequences using tBLASTn with the following settings: e value of 10^−5^, alignment length > 70% and match identity > 60%. Whole-genome BLASTp comparisons were performed between *H. parasuis* and *Actinobacillus pleuroneumoniae* serovar 5b strain L20 [GenBank: NC_009053.1], using the following settings: e value of 10^−5^, alignment length > 90% and match identity > 40%. All transcriptomic data were deposited in the Gene Expression Omnibus database [GEO: GSE56428].

## Results

### Biofilm formation under static conditions

**Figure 1:**
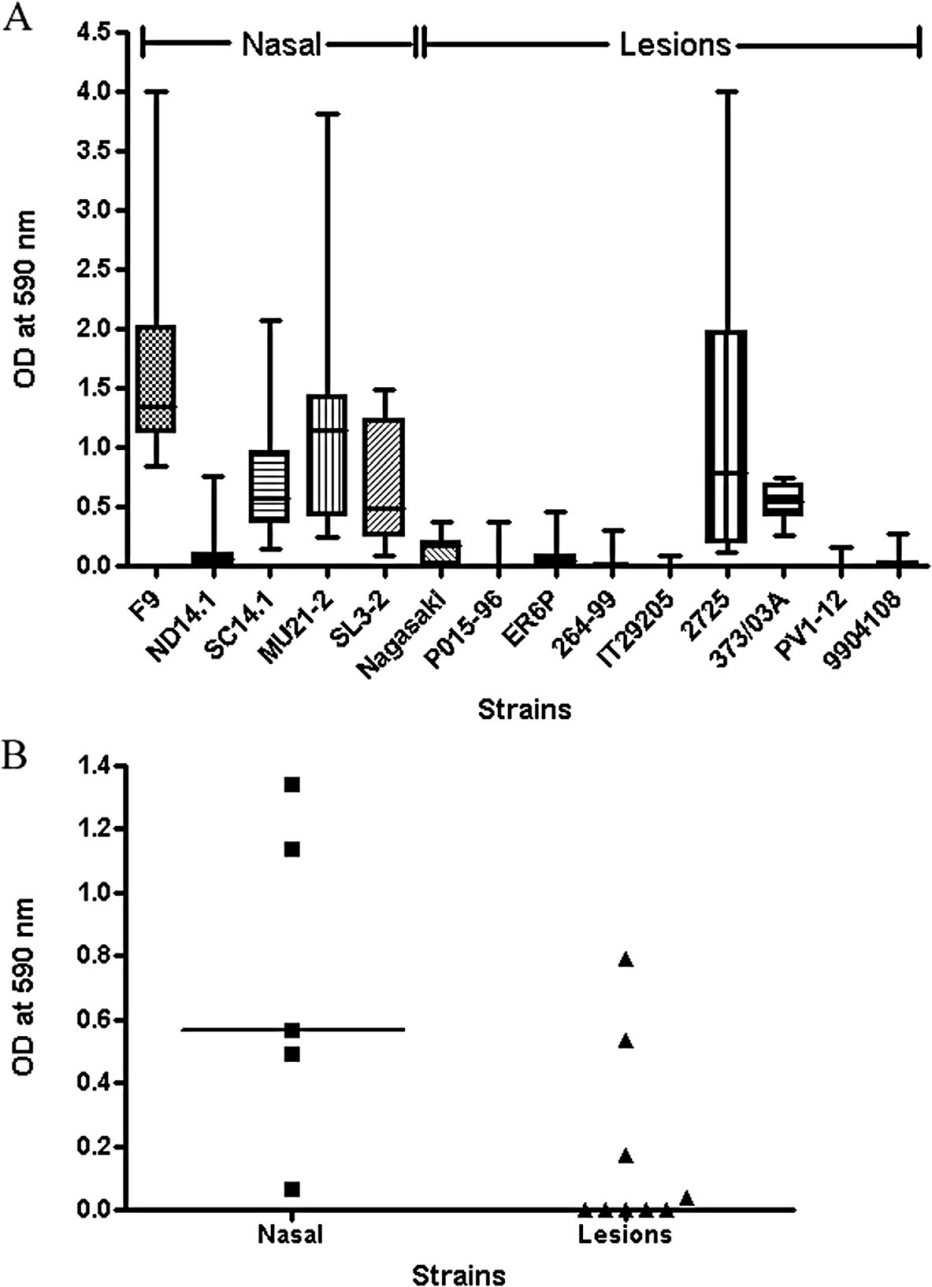
**Biofilm formation by** ***H. parasuis*** **isolates. (A)** Biofilm formation under static conditions in microtiter plates for *Haemophilus parasuis* nasal strains (*n* = 5) and strains isolated from lesions of pigs with Glässer’s disease (*n* = 9). **(B)** Medians of biofilm formation for *H. parasuis* strains isolated from the nasal cavities of healthy pigs (*n* = 5) or for strains isolated from the lesions of pigs with Glasser’s disease (*n* = 9). Difference between the median of the two groups of strains was statistically significant (*p* < 0.05).

The phenotypic and genotypic characteristics of the *H. parasuis* strains used in the present study are given in Table 1. Five non-virulent strains were recovered from the nasal cavities of healthy pigs while nine virulent strains were recovered from lesions of pigs with Glässer’s disease. The ability of *H. parasuis* to form biofilms at the solid–liquid interface in polystyrene microtiter plates was determined for each of the virulent and non-virulent strains (Figure 1A). Interestingly, the nasal, non-virulent strains formed significantly (*p* < 0.05) more biofilms than the virulent strains isolated from lesions of pigs with Glässer’s disease (Figure 1B). We also noticed that biofilm production was stronger in strains sensitive to serum compared to resistant strains (Additional file 1A, *p* = 0.059); in strains negative for *vtaA* group 1 genes compared to strains positive for these genes (Additional file 1B, *p* = 0.189); in strains belonging to MLST cluster C compared to strains belonging to MLST cluster A (Additional file 1C, *p* = 0.202), and in strains negative for the sialyltransferase gene *lsgB* compared to strains positive for *lsgB* (Additional file 1D, *p* = 0.228). These differences however were not statistically significant probably due to the small number of strains in some of the groups that were compared.

**Figure 2:**
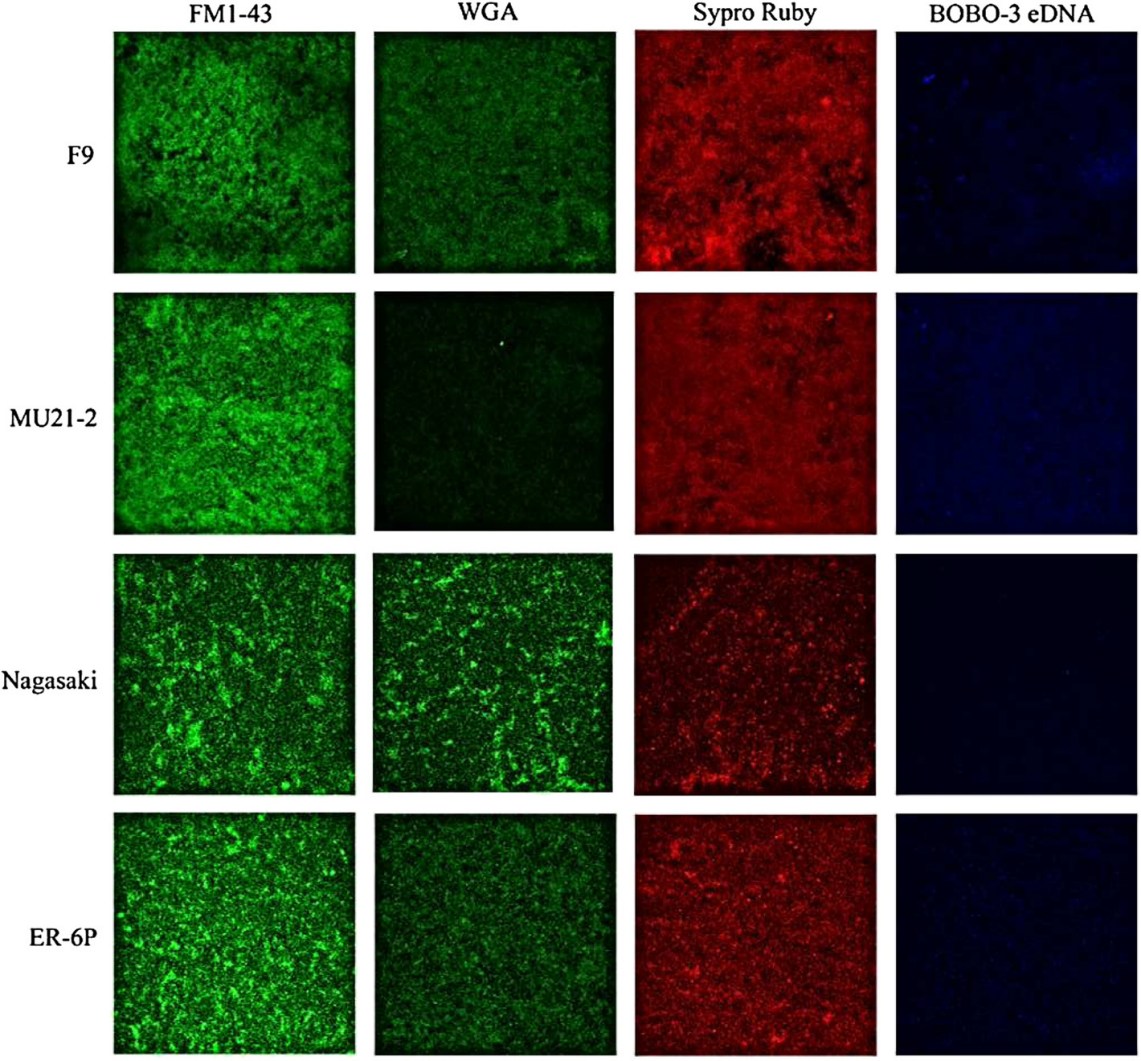
**Images of** ***H. parasuis*** **biofilms obtained by CLSM.** Confocal laser scanning microscopy of *Haemophilus parasuis* strains F9, MU21-2, Nagasaki and ER-6P biofilms formed under static conditions in wells of microtiter plates. Biofilms were stained with FilmTracer^™^ FM 1–43, wheat-germ agglutinin (WGA)-Oregon green 488 (for poly-*N*-acetyl glucosamine), SYPRO Ruby (for proteins) and BOBO-3 (for eDNA).

**Figure 3:**
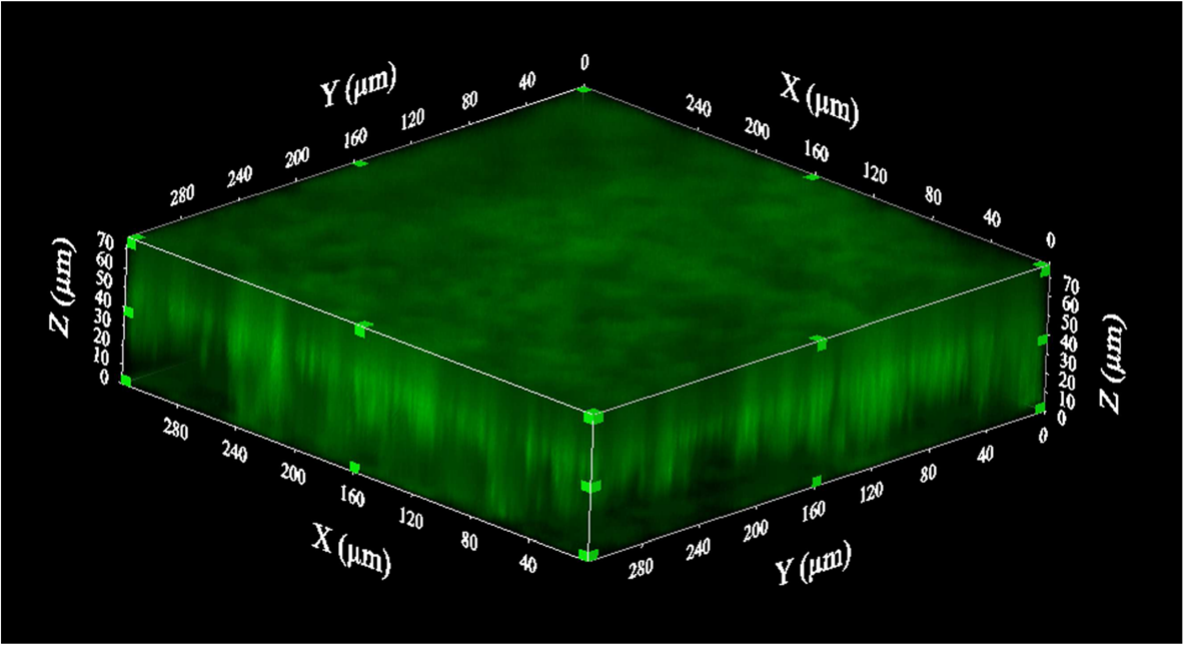
**3D images of** ***H. parasuis*** **strain F9 obtained by CLSM.** Confocal laser scanning microscopy three-dimensional images of *Haemophilus parasuis* strain F9 biofilm formed under static conditions in wells of a microtiter plate. Biofilm was stained with wheat-germ agglutinin (WGA)-Oregon green 488. Stack of sections of the X-Z plane of the biofilm.

Confocal laser scanning microscopy using different fluorescent probes was performed with the nasal, high-biofilm producer strains F9 and MU21-2 and the weak-biofilm producer virulent strains Nagasaki and ER-6P. The biofilm cells were stained with FilmTracer™FM®1-43, which becomes fluorescent once it is inserted in the cell membrane. The biofilms were also stained with fluorescent probes to label poly-N-acetylglucosamine (PGA), proteins, or extracellular DNA that could be present in the biofilm matrix. The data indicated that the biofilm matrix of these strains contains PGA, proteins, and eDNA; strain MU21-2 seemed however to produce less PGA than the other three strains (Figure 2). To further characterize the biofilms, 15 images of biofilm layers were recorded and stacked, and 3D-images of the biofilms were generated (Figure 3). Based on these reconstructions, the thickness as well as the biomass of the biofilm produced by each strain was evaluated. The thickness of the weak-biofilm producer strains Nagasaki and ER-6P was approximately 40 μm while the thickness of the high-biofilm producer strains MU21-2 and F9 was 50 μm and 70 μm, respectively. This was in agreement with the biomass (μm^3^/μm^2^) which was 17 for the weak-biofilm producer strain Nagasaki while the biomass of the high-biofilm producer strain F9 was 34.

**Figure 4:**
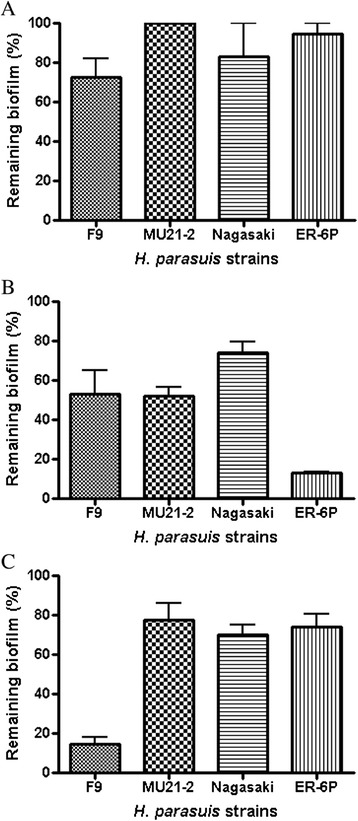
**Effect of enzymatic treatments on** ***H. parasuis*** **biofilms.** Dispersion of *Haemophilus parasuis* biofilms formed under static conditions in microtiter plates by **(A)** dispersin B, **(B)** proteinase K, and **(C)** DNase I.

Biofilms of strains F9, MU21-2, Nagasaki and ER-6P were digested with enzymes to further characterize the composition of the matrix. All strains were resistant to dispersin B (more than 70% of the biofilm remained after treatment) (Figure 4A). All strains were however sensitive to the proteinase K treatment (Figure 4B), with strain Nagasaki showing the lowest sensitivity (~80% of the biofilm remaining after treatment) and strain ER-6P showing the highest sensitivity (less than 20% of the biofilm remaining after treatment). Most strains were not affected by DNase I treatment (more than 70% of the biofilm remained after treatment) except strain F9 which was highly sensitive (with less than 20% of the biofilm remaining after treatment) (Figure 4C). This suggested that proteins, and, in one strain, extracellular DNA play a larger role than PGA in *H. parasuis* biofilm formation.

### Biofilm formation under shear force conditions

**Figure 5:**
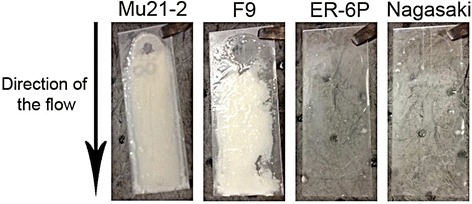
**Biofilm formation by** ***H. parasuis*** **in a drip-flow apparatus.** Biofilm formation under low shear force in a drip-flow apparatus. Images of typical biofilms for *Haemophilus parasuis* strains MU21-2, F9, ER-6P, and Nagasaki visible after 24 h of incubation with continuous flow (25 mL/h).

Previous experiments with *A. pleuropneumoniae* and the drip-flow apparatus indicated that this biofilm reactor could be used to grow biofilm of fastidious *Pasteurellaceae* isolated from the upper respiratory tract [24]. The conditions (50 mL/channel/hour; 50% BHI-NAD) used to grow *A. pleuropneumoniae* biofilms in the drip-flow apparatus were used for the first test. However, these did not support the growth of *H. parasuis* biofilms. Given that *H. parasuis* is more fastidious than *A. pleuropneumoniae*, a full strength BHI-NAD was used and the speed of the flow was reduced by half. These conditions allowed the two strains with a strong-biofilm phenotype, MU21-2 and F9, to produce visible biofilms (Figure 5). Although a thin film was observable for Nagasaki and ER-6P, which are strains with a weak-biofilm phenotype under static conditions, the conditions of the drip-flow apparatus did not enhance the biofilm formation by these strains (Figure 5). As indicated by the biofilm dry weight, strain MU21-2 produced a larger biomass than strain F9 under these flow conditions (Table 2).

**Table 2:** **Average dry weight (in mg) for drip-flow biofilms formed after 24 h of continuous flow by 4 different strains of** ***Haemophilus parasuis***

**Strain**	**Biofilm dry weight (±Standard error of the mean)**
MU21-2	8.33 (±3.23)
F9	3.37 (±0.38)
Nagasaki	ND^1^
ER-6P	ND

^1^The amount of biofilm was below the detection limit.

**Figure 6:**
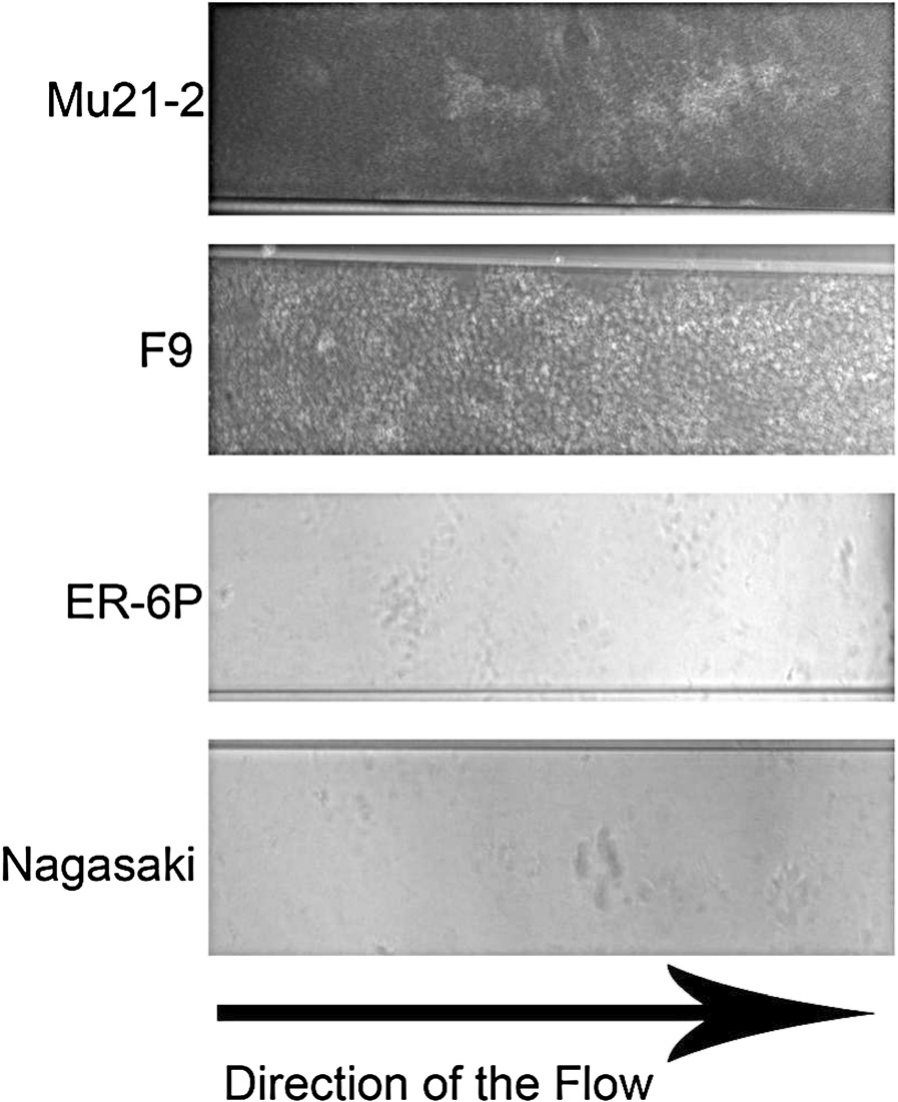
**Biofilm formation by** ***H. parasuis*** **in a microfluidic system.** Biofilm formation under controlled shear force in a BioFlux 200 microfluidic system. Phase-contrast images of typical biofilms of *Haemophilus parasuis* non-virulent strains MU21-2 and F9, and virulent strains ER-6P and Nagasaki obtained after 24 h of incubation with an inoculum of OD_600_ of 0.25 and a shear force of 0.5 dyne/cm^2^.

The BioFlux 200 flowthrough device is a high throughput microfluidic system that has been recently tested for the growth of bacterial biofilms [25]. This system has yet to be tested with *Pasteurellaceae* and could provide certain advantages over the drip-flow apparatus. For example, the system requires smaller volumes, which range in μL to mL, and can be used for high throughput screens. Initial density of the inoculum (OD_600_ of 1.0, 0.5 and 0.25) and the incubation time (2 h and 4 h) for the initial attachment were the first parameters tested with a relatively low shear force (0.5 dyne/cm^2^). An OD_600_ of 0.25 and an incubation time of 2 h for the initial attachment were sufficient for strains MU21-2 and F9 to form biofilms that block the microfluidic channel after 6 h (Figure 6); however, strains Nagasaki and ER-6P did not form biofilms (Figure 6). To prevent blocking of the channel, a range of shear force was tested with an inoculum at OD_600_ of 0.25 and an incubation time of 2 h for the initial attachment. Both strains MU21-2 and F9 were not able to form a biofilm when the shear force was equal or above 0.7 dyne/cm^2^. Between 0.5 and 0.7 dyne/cm^2^, both strains always blocked the channel within 12 h but it took longer as the shear force was increased. Therefore, in our hands, this microfluidic system can only be used to study biofilm formation of *H. parasuis* during short incubation periods.

### Effect of fibrinogen and fibronectin on biofilm formation

**Figure 7:**
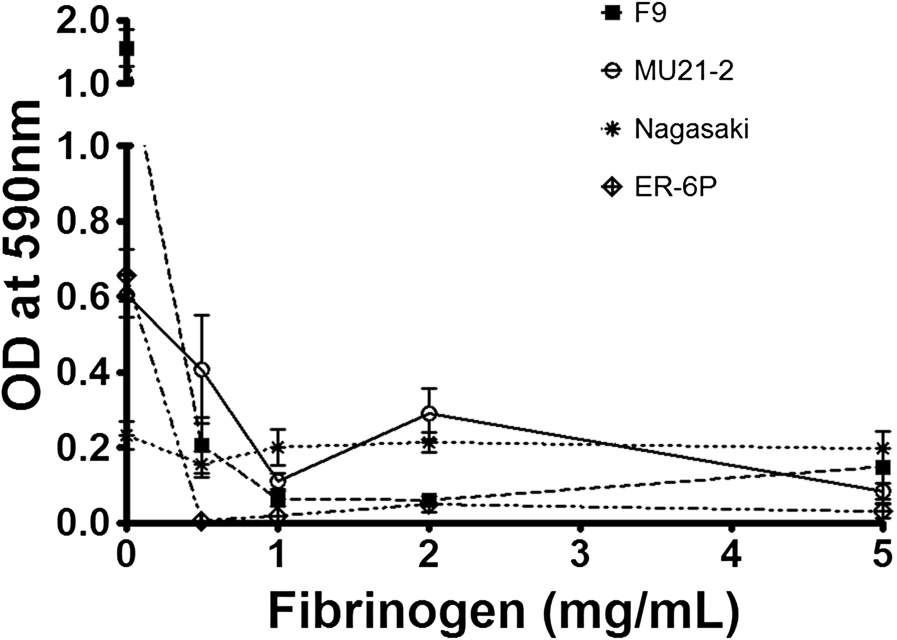
**Effects of fibrinogen on biofilm formation by** ***H. parasuis*** **.** Effects of various concentrations of fibrinogen added to the culture medium on biofilm formation by *Haemophilus parasuis* strains F9, MU21-2, Nagasaki and ER-6P under static conditions in microtiter plates. Assays were performed in triplicate, and the means ± standard deviations are indicated.

It has been shown that supplementing the culture medium with fibrinogen induces biofilm formation of *Streptococcus suis*, another important swine pathogen [46]. Thus, we evaluated the effect of supplemental fibrinogen on *H. parasuis* biofilm formation. As shown in Figure 7, fibrinogen (at a concentration of 1 mg/mL) inhibited biofilm formation by all four strains. Fibronectin had no effect on bioflm formation (data not shown). Inhibition of biofilm formation by fibrinogen was not related to an inhibition of growth since fibrinogen did not affect growth of *H. parasuis* (data not shown).

### Transcriptional profile of *H. parasuis* F9 grown in biofilm

**Figure 8:**
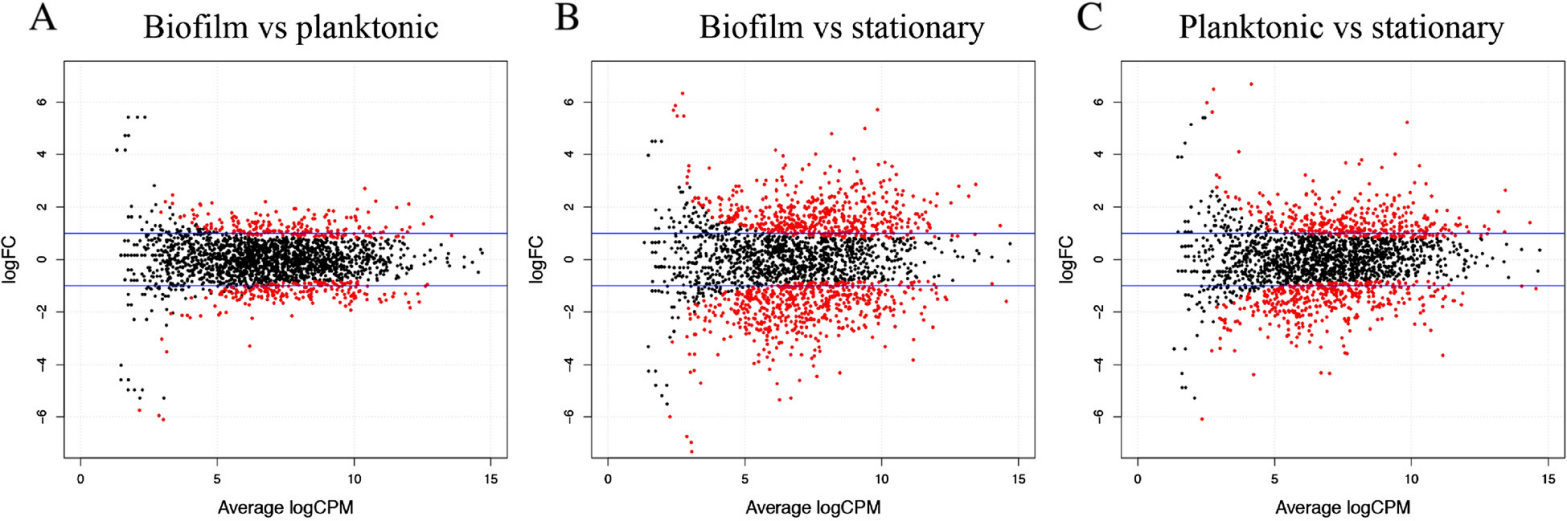
**Identification of** ***H. parasuis*** **genes differentially expressed.** MA plots generated by EdgeR showing transcript expression profiles in the three comparisons performed: biofilm vs planktonic **(A)**, biofilm vs stationary phase **(B)** and planktonic vs stationary phase **(C)**. For each gene, log_2_(fold change) between the two conditions is plotted (M, y axis) against the gene’s log_2_(average expression) in the two samples (A, x axis). The blue lines indicate 2-fold changes. Red dots highlight the genes at 5% *P*-value.

**Figure 9:**
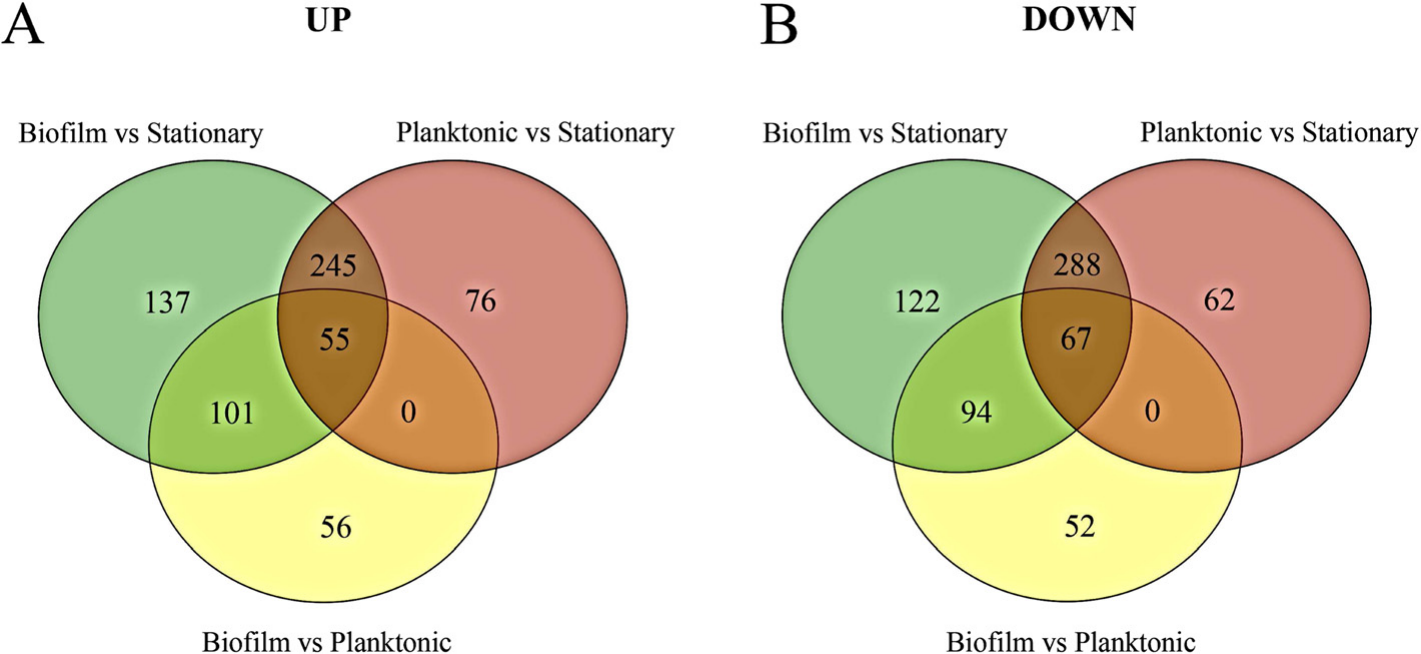
**Comparison of** ***H. parasuis*** **genes that were up- or down-regulated.** Venn diagrams of *Haemophilus parasuis* genes identified as up**- (A)** and down-regulated **(B)** under different growth states.

Given that the genome of strain F9 was not previously sequenced, the genome of this high-biofilm producer was sequenced to facilitate transcriptional analysis. The assembly size of the F9 genome was 2.49 Mb, with an n50 of 44 023 and 644 contigs, with a G + C content of 39%, which is comparable to that of the draft and complete genomes of *H. parasuis* [30,47]. Transcriptomic analysis was performed with an average of 2 M sequence reads per mRNA sample. More than 75% of the reads for each sample were mapped. The majority of reads mapped with a mapping quality (MAPQ) ≥ 20, and only reads mapping with a MAPQ ≥ 10 were further processed for differential gene expression analysis (Additional file 2A). Some reads mapped in non-protein coding sequences, mainly in tRNA gene sequences, and were particularly high in the stationary culture sample (Additional file 2B). Differential expression analysis found 425 DEGs in biofilm (B) when compared to planktonic growth (P) (Table 3). When B or P condition was compared to the stationary-phase culture (S), a notable increase in the number of up-regulated genes was observed (Figure 8 and Table 3). Filtered lists of DEGs for B vs P, P vs S and B vs S are shown in Additional files 3, 4 and 5, respectively. A large number of up- and down-regulated genes were shared between B vs S and P vs S comparisons (Figure 9A and B), although a considerable amount was unique to each condition. Fifty-five genes were up-regulated in all three comparisons, which included 8 ribosomal proteins. On the other hand, 56 up-regulated genes were unique to the biofilm and included, among others, six transcriptional regulators, possibly involved in biofilm formation (Additional file 6).

**Table 3:** **Summary of the differential expression analysis performed with edgeR tool (** ***P*** **-value < 0.05)**

**Comparison**	**Up**	**Down**	**Total**	^**4**^ **Genome (%)**
^1^B vs ^2^P	212	213	425	19
B vs ^3^S	538	571	1109	49
P vs S	376	417	793	35

^1^Biofilm.

^2^Planktonic.

^3^Stationary phase.

^4^2259 of the 2317 annotated protein-coding genes were taken as total (at least one count per million (cpm) in at least two samples).

Percentages of differentially expressed genes are also shown for each comparison.

Blast2GO allowed 76% of GO term assignment to *H. parasuis* proteome, with a good GO level distribution (mean level = 6.8; SD = 2.7) and more than 8 K annotations. Enrichment analysis of the DEGs (*P* < 0.05) identified a large number of up- or down-regulated pathways (Additional file 7). Analysis was visualized performing Venn diagrams (Additional files 8 and 9). All three comparisons shared the enriched up-regulated GO term “structural constituent of ribosome”. Enrichment of B vs S and P vs S shared the following GO terms: activation of response to oxidative stress, iron ion binding, ribosome biogenesis, unfolded protein binding and peroxidase activity pathways, indicating the importance of these functions for both biofilm and planktonic growth. Interestingly, protein folding, cell outer membrane, protein secretion, and sequence-specific DNA binding (transcriptional regulators) GO terms were up-regulated specifically in B vs S and B vs P comparisons, which could indicate specific biofilm pathways. Other GO terms were specific to each comparison. A total of 20 up-regulated GO terms were specific to B vs P comparison, such as lipopolysaccharide transport, response to stress or DNA-mediated regulation of transcription (transcriptional regulators) (Additional file 9). The same analysis was performed for down-regulated genes and showed that membrane-related genes were over-represented among these genes in all three comparisons. These membrane-related genes were related to transport, more especially to sodium ion transport or phosphoenolpyruvate-dependent sugar phosphotransferase system (PTS). B vs S and P vs S detected down-regulated genes related to nutrient transport, such as ions, amino acids or monosaccharides, which indicate common pathways in biofilm and planktonic conditions. On the other hand, some enriched GO terms were specific to biofilm, as evidenced by the B vs P comparisons. For instance, GOs related to translation (ribosomal proteins) were among the most significantly down-regulated, which suggest a possible low metabolic state, but these enriched GO terms were caused by different ribosomal proteins than the ones responsible for the shared enriched up-regulated GO term (Additional file 9).

The F9 surface-associated proteins were predicted using Phobius and the conservation of the up-regulated ones was examined by tBLASTn in the 14 *H. parasuis* genomic sequences available in GenBank. The number of predicted membrane proteins up-regulated in F9 grown under biofilm conditions (B vs S) was 148 from a total of 538. The majority of the predicted membrane proteins were well conserved among the majority of strains, including a 28 kDa outer membrane protein (HS372_00711), Omp P5 precursor (HS372_01222), PilQ (HS372_02002) and Omp85 (HS372_00165) (Additional file 10). Others were found exclusively in non-virulent isolates, such as OmpW (HS372_00498), prophage CP4-57 integrase (HS372_00596), filamentous hemagglutinin FhaB (HS372_01074) or five hypothetical proteins (HS372_00147, HS372_01332, HS372_02390, HS372_00611 and HS372_02391) (Additional file 10). Additionally, FhaC (annotated as TpsB) was also found only in the non-virulent strains (Additional file 10).

*H. parasuis* gene expression during biofilm and planktonic growth was compared to transcriptomic data of *A. pleuropneumoniae*, another swine pathogen member of the *Pasteurellaceae* family, from a previous study [24]. Although preliminary analysis showed that *A. pleuropneumoniae* and *H. parasuis* F9 shared only 60% of the genome, some common up-regulated genes were found under biofilm condition (Table 4), but no predominant pathways were detected, suggesting different regulatory networks for these two species. Notably, proteins related to anaerobic metabolism such as cytochrome c-type protein NapC (HS372_02085) or putative electron transport protein yccM (HS372_02091) and some lipoproteins (HS372_01222 and HS372_00366) were found in both bacteria.

**Table 4:** **Common up-regulated genes between** ***Haemophilus parasuis*** **F9 and** ***Actinobacillus pleuropneumoniae*** **when grown in biofilm condition**

***H. parasuis*** **(this study) vs** ***A. pleuropneumoniae*** **[** 22 **]**		
***H. parasuis*** **F9**	**App**	**Product**
HS372_02083	APL_1425	Cytochrome c-type protein NapC
HS372_02085	APL_1427	Putative electron transport protein yccM
HS372_02091	APL_1821	50S ribosomal protein L31 type B
HS372_00945	APL_1440	High-affinity zinc uptake system protein znuA precursor
HS372_00147	APL_1894	hypothetical protein
HS372_02009	APL_1423	Putative esterase
HS372_02012	APL_0433	Peptide methionine sulfoxide reductase MsrB
HS372_01222	APL_0460	Outer membrane protein P5 precursor
HS372_00060	APL_1206	putative ribonuclease FitB
HS372_01900	APL_1173	Nicotinamide riboside transporter pnuC
HS372_00666	APL_0442	vancomycin high temperature exclusion protein
HS372_01587	APL_0484	Alpha-aminoadipate--lysW ligase lysX
HS372_02062	APL_0038	hypothetical protein
HS372_00364	APL_0222	Putative lipoprotein/NMB1162 precursor
HS372_01892	APL_0133	Cys regulon transcriptional activator
HS372_00988	APL_1295	Arginine repressor
HS372_02252	APL_1873	Succinyl-diaminopimelate desuccinylase
HS372_02064	APL_0036	hypothetical protein
HS372_01893	APL_0134	hypothetical protein
HS372_01521	APL_1320	Thiamine import ATP-binding protein ThiQ
HS372_02387	APL_1059	Integrase core domain protein
HS372_00061	APL_1207	prevent-host-death family protein
HS372_00916	APL_0423	Ribonuclease HI
HS372_01200	APL_0593	Inosine-5’-monophosphate dehydrogenase
HS372_01950	APL_1574	Putative hydrolase ydeN
HS372_02244	APL_0254	Cytosol non-specific dipeptidase
HS372_01281	APL_1230	Phosphoserine phosphatase
HS372_01385	APL_0967	Glutamate permease
HS372_01220	APL_0463	Putative phosphinothricin acetyltransferase YwnH
HS372_01490	APL_0928	hypothetical protein
HS372_01179	APL_1499	Threonine synthase
HS372_02366	APL_0395	Sigma-E factor negative regulatory protein
HS372_01208	APL_0895	Formate dehydrogenase-N subunit gamma
HS372_01221	APL_0461	putative phosphatase YwpJ
HS372_02265	APL_0687	D-lactate dehydrogenase
HS372_01342	APL_1448	Spermidine/putrescine import ATP-binding protein PotA
HS372_00366	APL_0220	Putative lipoprotein/NMB1164 precursor
HS372_00099	APL_1853	Ketol-acid reductoisomerase

## Discussion

In the present study, we used well-characterized virulent and non-virulent strains of *H. parasuis* from our collection [13]. We found that most nasal strains (4/5) were strong-biofilm producers whereas most systemic strains (8/9) formed no biofilm or were very weak-biofilm producers. Similarly, a strong association was found between MLST clusters [2] and biofilm formation. Strains of MLST cluster C (which are associated with nasal isolation) formed more biofilms than strains of MLST cluster A (which are isolated from systemic lesions). Similarly, the presence of autotransporter *vtaA* group 1 genes and of sialyltransferase *lsgB* gene associated with virulent strains [13,48], and was not associated with strong biofilm formation. Overall, our results are in agreement with Jin et al. [19] who observed that, generally, serovars considered non-virulent showed a higher degree of biofilm formation than virulent serovars. Although it is important to note that a strict relationship between serovars and virulence in *H. parasuis* has not been demonstrated [1].

The use of CLSM and fluorescent probes showed the presence of PGA, proteins, and eDNA in the biofilm matrix of *H. parasuis* nasal, high-biofilm producer strains F9 and MU21-2 and of the weak-biofilm producer strains Nagasaki and ER-6P recovered from lesions of pigs with Glässer’s disease. This is, to the best of our knowledge, the first report of the presence of PGA and proteins in the biofilm matrix of *H. parasuis*. PGA is the major component of the biofilm matrix of several bacteria including other *Pasteurellaceae* members such as *A. pleuropneumoniae* and *Aggregatibacter actinomycetemcomitans* [18,49]. Nevertheless all 4 strains tested were resistant to dispersin B. All strains were however sensitive to a proteinase K treatment suggesting that proteins play a larger role than PGA in *H. parasuis* biofilm formation. Tang et al. [50] have shown that treatment with the staphylococcal nuclease NUC1 decreased slightly the biofilm formation of *H. parasuis* strain 0322. This is in agreement with results of this study showing the presence of eDNA in the biofilm matrix of *H. parasuis* and the high sensitivity of strain F9 biofilm to DNase I digestion.

This is also the first description of *H. parasuis* biofilm formation under shear force in a drip-flow reactor and a microfluidic system. Interestingly, similar biofilm phenotypes (i.e. high-producer vs weak-producer) were observed when static or controlled flow conditions were used. The drip-flow apparatus is a system that it is thought to create an environment with an air-liquid interface which closely resembles the lung environment [23]. Under the conditions we selected, the two strains with a strong-biofilm phenotype, MU21-2 and F9, produced visible biofilms while a thin film was observable for Nagasaki and ER-6P, which are strains with a weak-biofilm phenotype under static conditions. We also used the BioFlux flowthrough device, a high throughput microfluidic system that has been recently tested for the growth of dental plaque bacteria biofilms [51] and *Pseudomonas aeruginosas* biofilms [25]. Again, strains Nagasaki and ER-6P did not form biofilms under the conditions tested but strains MU21-2 and F9 formed biofilms that rapidly blocked the microfluidic channel. This microfluidic system can therefore be used to study biofilm formation of *H. parasuis* but for short incubation periods. This potentially can be used to investigate genes involved in the early steps of biofilm formation and, if the appropriate tools are combined, to study real-time gene expression during the early steps as demonstrated in *Staphylococcus aureus* [52].

The genes involved in *H. parasuis* biofilm formation are currently not known. Recently, it was reported that both *galU* and *galE* genes seemed to play a role in biofilm formation of *H. parasuis* [12]. A *galU* mutant was unable to form biofilm in a glass tube while a *galE* mutant produced more biofilm than the parent wild-type strain. Surprisingly however, the *galU* mutant also showed an increased tendency to autoagglutinate which is usually associated with a greater ability to form biofilm. Here, the transcriptome of *H. parasuis* F9 strain showed that static biofilm and planktonic cultures are in similar biological states but not identical, whereas greater differences in gene expression were evident when compared to stationary-phase culture. Subtle differences in gene expression between biofilm and planktonic cells have been also reported for other bacterial species [53,54]. In addition to identifying DEGs related to metabolism, gene enrichment allowed the identification of a large proportion of membrane-related genes among the up-regulated genes in biofilm, including some that have been reported for other bacterial species [53,54]. The conservation of some highly expressed membrane-protein genes in biofilms among the 14 *H. parasuis* genomes available indicates that those may not specifically associate with biofilm formation. A sub-set of these up-regulated membrane-protein genes, such as the type IV pilus biogenesis protein *pilQ*, may be involved in adhesion to different abiotic and biotic surfaces. Interestingly, some up-regulated genes, such as *fhaB* and *fhaC* (*tpsB*) or *ompW*, were only found in the genome of non-virulent strains. This finding, together with the fact that non-virulent strains formed stronger biofilms, suggests a possible role of these genes in biofilm formation. In fact, Fha and OmpW, together with type IV pilus, have been shown to play a role in biofilm formation in other bacteria [55,56]. Additionally, some lipoproteins, such as lipoprotein Plp4, as well as signs of anaerobic metabolism were evidenced in the biofilms of *H. parasuis* and *A. pleuropneumoniae*, which may indicate a potential role in biofilm formation of both bacterial species. The function of specific genes in biofilm formation will need further confirmation.

Interestingly, experimental infections in snatch-farrowed, colostrum-deprived piglets showed that the *H. parasuis* strains that proceeded to invade the host were not maintained well in the nasal cavities of the piglets [57]. This suggests changes in the bacteria from a “colonizing state” to an “invasive state”, which could be modulated by the ability of each animal to control the infection. In the light of the results of the present study, these changes from a “colonizing state” to an “invasive state” could also be due to the ability of a given bacterial strain to form or not a robust biofilm. We propose that biofilm formation might allow the non-virulent strains to colonize and persist in the upper respiratory tract of pigs. Conversely, the predominant planktonic state of the virulent strains might allow them to disseminate within the host. This latter statement is supported by the inhibition of biofilm formation by fibrinogen. It is worth noting that a recent study on the human pathogen *Streptococcus pneumoniae* showed that biofilm formation in vivo is associated with reduced invasiveness and a dampened cytokine response [58]. High-biofilm production phenotype might therefore not always be linked to virulence.

## Additional files

Additional file 1:
***Haemophilus parasuis*** **biofilm formation under static conditions in microtiter plates. (A)** Medians of biofilm formation for strains that are sensitive or show intermediate resistance to serum (*n* = 9) or for strains that are resistant to serum (*n* = 5). **(B)** Medians of biofilm formation for strains negative (*n* = 5) or positive (*n* = 9) for *vtaA* group 1 genes. **(C)** Medians of biofilm formation for strains belonging to MLST cluster C (*n* = 5) or strains belonging to MLST cluster A (*n* = 7). **(D)** Medians of biofilm formation for strains negative (*n* = 8) or positive (*n* = 6) for the sialyltransferase gene *lsgB*. Differences between the median of the two groups of strains were not statistically significant (**A**: *p* = 0.059; **B**: *p* = 0.189; **C**: *p* = 0.202; **D**: *p* = 0.228). Additional file 2:
**Mapping of RNA sequencing reads to the** ***H. parasuis*** strain F9 genome. Overview of the mapping **(A)** and read counts **(B)** results. Mapping quality (MAPQ) shows that most of the reads were aligned with MAPQ ≥ 20 but a considerable percentage of reads were not taken into account for differential expression because mapping to non-protein coding regions, particularly for stationary culture sample. Additional file 3:
**List of** ***H. parasuis*** **strain F9 genes differentially expressed in biofilm vs planktonic cells.** Differentially expressed genes in biofilm versus planktonic *H. parasuis* cells (FDR < 0.05). Sorted by logFC. *Actinobacillus pleuropneumoniae* (App) first BLASTp hits are also shown. Additional file 4:
**List of** ***H. parasuis*** **strain F9 genes differentially expressed in planktonic vs stationary phase cells.** Differentially expressed genes in planktonic versus stationary *H. parasuis* cells (FDR < 0.05). Sorted by logFC. *Actinobacillus pleuropneumoniae* (App) first BLASTp hits are also shown. Additional file 5:
**List of** ***H. parasuis*** **strain F9 genes differentially expressed in biofilm vs stationary phase cells.** Differentially expressed genes in biofilm versus stationary *H. parasuis* cells (FDR < 0.05). Sorted by logFC. *Actinobacillus pleuropneumoniae* (App) first BLASTp hits are also shown. Additional file 6:
**List of** ***H. parasuis*** **strain F9 up-regulated genes or unique to biofilm.** *Haemophilus parasuis* F9 genes up-regulated in all three comparisons (biofilm vs planktonic; biofilm vs stationary phase; planktonic vs stationary phase) or unique to biofilm. Additional file 7:
**List of gene ontology (GO) terms following enrichment analysis of** ***H. parasuis*** **strain F9 differentially expressed genes.** Enriched Gene Ontology biological process and cellular component nodes among up-regulated genes (FDR < 0.001). No enriched CC GO terms were found for biofilm vs planktonic comparison. Additional file 8:
**Visualization of gene ontology (GO) terms following enrichment analysis of** ***H. parasuis*** **strain F9 differentially expressed genes.** Venn diagrams of *Haemophilus parasuis* enriched Gene Ontology (GO) terms among the subsets of differentially expressed genes identified as up- **(A)** and down-regulated **(B)** under different growth states. Only most specific GO terms are shown. Additional file 9:
**List of** ***H. parasuis*** **strain F9 GO terms shown in Additional file** 8 **diagrams.** *H. parasuis* F9 GO terms results from Additional file 8 Venn diagrams. Additional file 10:
**List of** ***H. parasuis*** **strain F9 membrane-related genes differentially expressed in biofilm compared to planktonic or stationary phase cells.** Conservation of *Haemophilus parasuis* F9 membrane-related genes differentially expressed (*P* < 0.05) in biofilms vs stationary culture or biofilms vs planktonic culture among 14 *H. parasuis* isolates. Genes are sorted by logFC. *Truncated.
