# Supplementary figures and images for: Biofilm formation by virulent and non-virulent strains of Haemophilus parasuis
Source: Vet Res. 2014 Nov 27;45(1):104. doi: 10.1186/s13567-014-0104-9 (PMC4245831; doi:10.1186/s13567-014-0104-9)

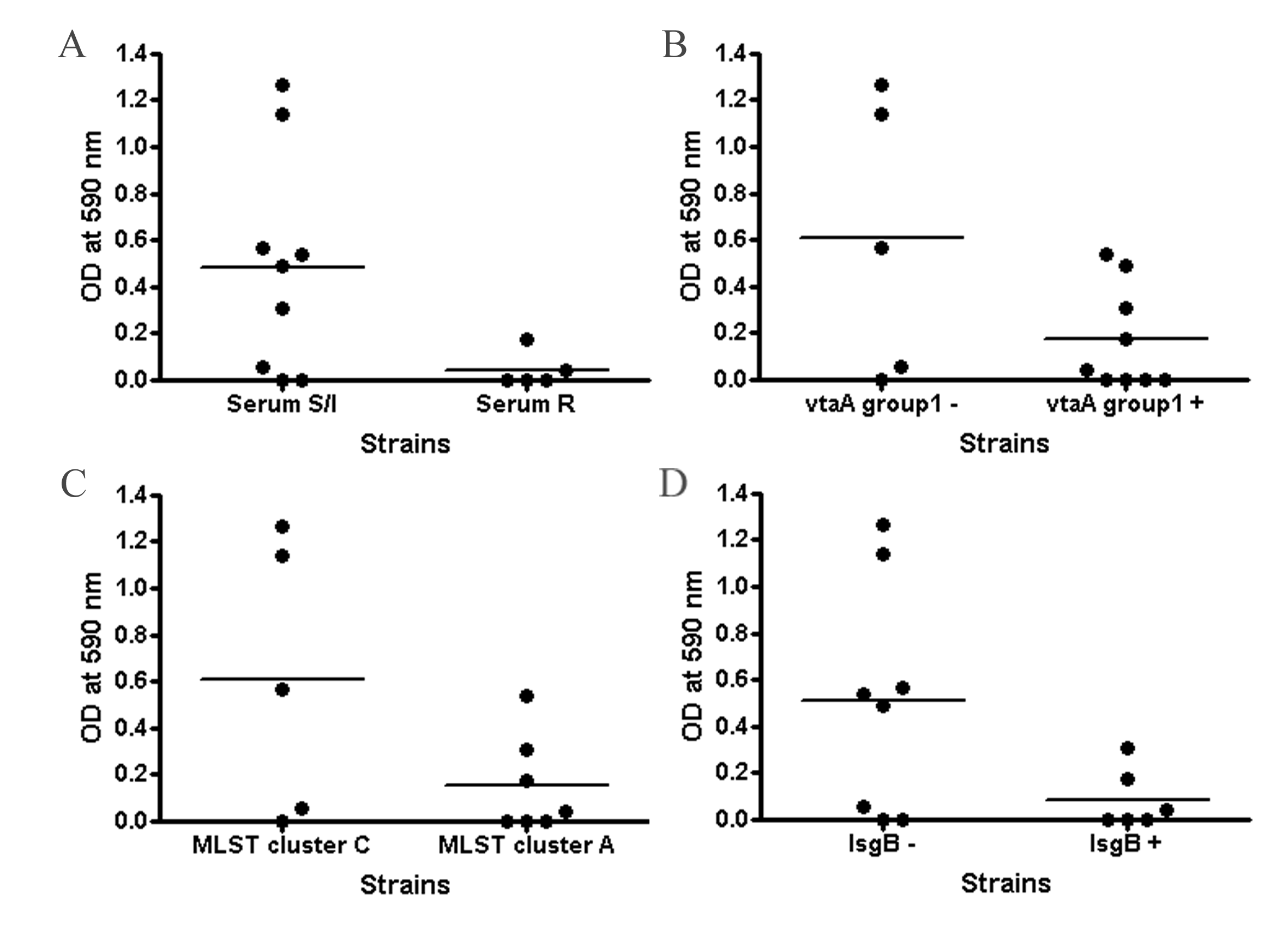

Supplement: Additional file 1: — Haemophilus parasuis biofilm formation under static conditions in microtiter plates. (A) Medians of biofilm formation for strains that are sensitive or show intermediate resistance to serum (n = 9) or for strains that are resistant to serum (n = 5). (B) Medians of biofilm formation for strains negative (n = 5) or positive (n = 9) for vtaA group 1 genes. (C) Medians of biofilm formation for strains belonging to MLST cluster C (n = 5) or strains belonging to MLST cluster A (n = 7). (D) Medians of biofilm formation for strains negative (n = 8) or positive (n = 6) for the sialyltransferase gene lsgB. Differences between the median of the two groups of strains were not statistically significant (A: p = 0.059; B: p = 0.189; C: p = 0.202; D: p = 0.228). [file 13567_2014_104_MOESM1_ESM.tiff]

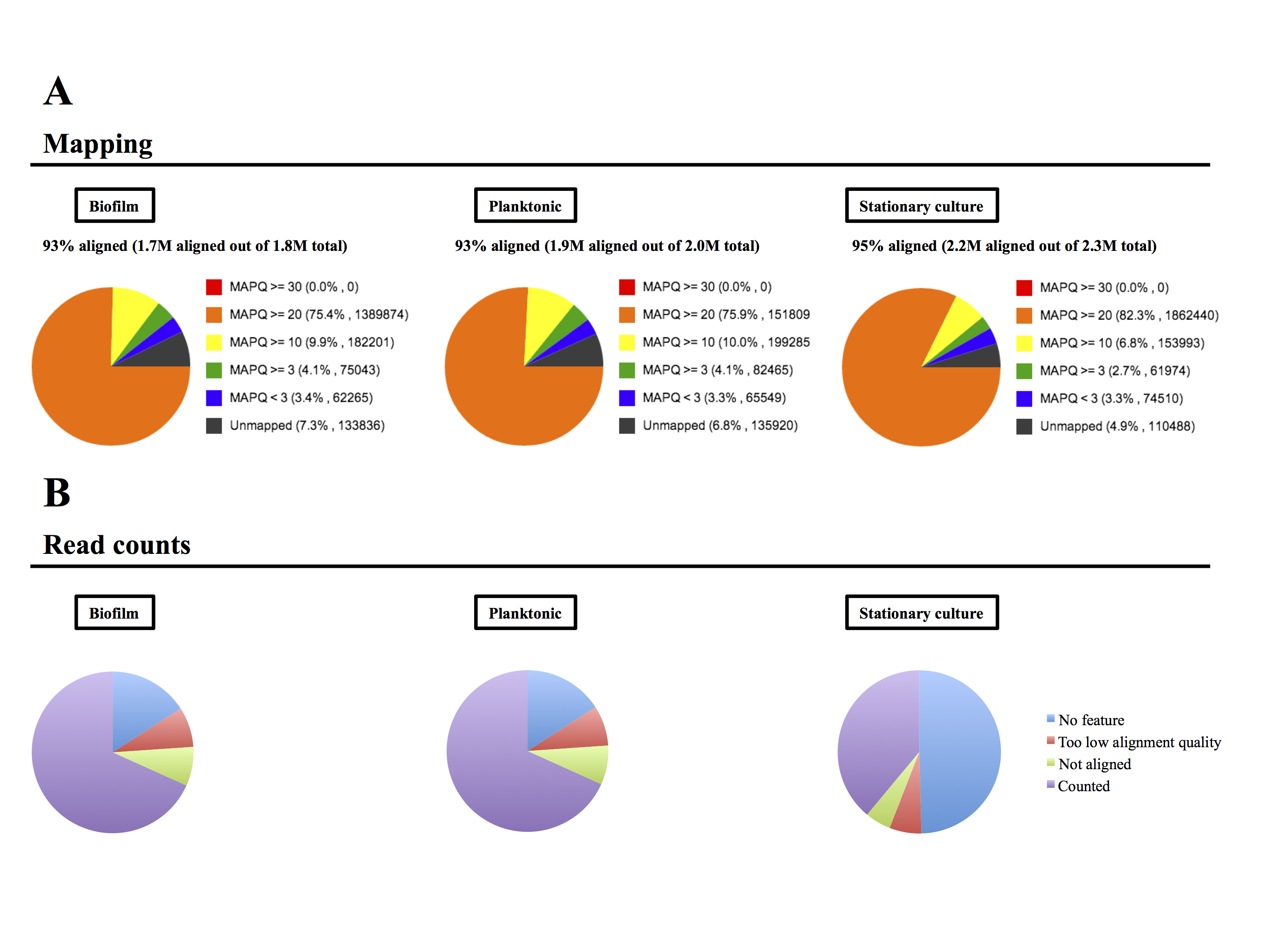

Supplement: Additional file 2: — Mapping of RNA sequencing reads to the H. parasuis strain F9 genome. Overview of the mapping (A) and read counts (B) results. Mapping quality (MAPQ) shows that most of the reads were aligned with MAPQ ≥ 20 but a considerable percentage of reads were not taken into account for differential expression because mapping to non-protein coding regions, particularly for stationary culture sample. [file 13567_2014_104_MOESM2_ESM.tiff]

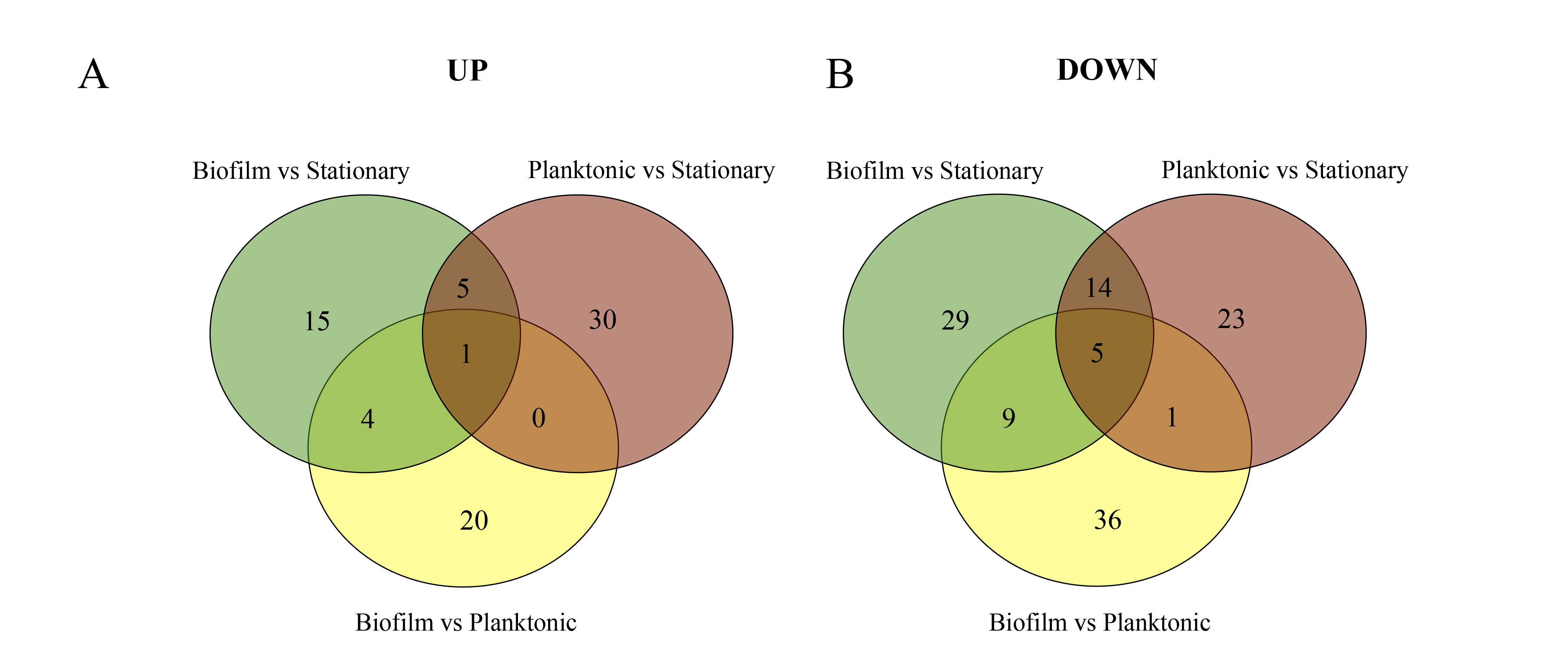

Supplement: Additional file 8: — Visualization of gene ontology (GO) terms following enrichment analysis of H. parasuis strain F9 differentially expressed genes. Venn diagrams of Haemophilus parasuis enriched Gene Ontology (GO) terms among the subsets of differentially expressed genes identified as up- (A) and down-regulated (B) under different growth states. Only most specific GO terms are shown. [file 13567_2014_104_MOESM8_ESM.tiff]
